# Metaplastic breast cancer: a comparison between the most common histologies with poor immunohistochemistry factors

**DOI:** 10.1186/s12885-015-1079-2

**Published:** 2015-02-20

**Authors:** Salim Abraham Barquet-Muñoz, Silvia Patricia Villarreal-Colin, Luis Alonso Herrera-Montalvo, Ernesto Soto-Reyes, Carlos Pérez-Plasencia, Jaime Coronel-Martínez, Delia Pérez-Montiel, Rafael Vázquez-Romo, David Cantú de León

**Affiliations:** 1 Department of Breast Tumors, Instituto Nacional de Cancerología, Mexico City, Mexico; 2 Direction of Research, Instituto Nacional de Cancerología, Mexico City, Mexico; 3 Clinical Research, Instituto Nacional de Cancerología, Mexico City, Mexico; 4 Department of Surgical Pathology, Instituto Nacional de Cancerología, Mexico City, Mexico

**Keywords:** Breast, Cancer, Metaplastic, Ductal, Lobular, Triple-negative

## Abstract

**Background:**

Metaplastic carcinoma of the breast (MCB) is a rare histological type of breast cancer. This study aimed to determine whether MCB exhibits shorter overall survival (OS) and disease-free survival (DFS) compared with other histologies that are considered unfavorable.

**Methods:**

We retrospectively analyzed 157 clinical file records of the Mexico City-based National Institute of Cancerology and compared the clinical characteristics and treatment of 24 patients with MCB, 37 patients with triple-negative invasive lobular carcinoma (TN-ILC), 48 patients with high-grade invasive ductal carcinoma (HG-IDC), and 48 patients with triple-negative invasive ductal carcinoma (TN-IDC), paired by clinical stage and age. We performed a comparative analysis and analyzed OS and DFS using a log-rank test.

**Results:**

In patients with MCB, the 5-year DFS was 52.1% (mean, 48.52 months; 95%: 35.32-61.72), and the 5-year OS was 72.2% (mean, 59.77 months; 95% CI: 48.55-71.00). No differences were observed in the DFS of MCB compared with each of the other histologies (MCB vs. HG-IDC, *p* = 0.865; MCB vs. TN-IDC, *p* = 0.966, and MCB vs. TN-ILC, *p* = 0.132). Moreover, no differences were observed when comparing the OS of MCB with that of each of the other histologies (MCB vs. HG-IDC, *p* = 0.246; MCB vs. TN-IDC, *p* = 0.255, and MCB vs. TN-ILC, *p* = 0.387).

**Conclusions:**

Neither OS nor DFS differ between patients with MCB and those with other histologies with unfavorable immunohistochemical factors.

## Background

Breast cancer (BC) is the most common malignant neoplasia in women in Mexico in terms of incidence and mortality [1,2]. Approximately 85% of BC cases involve invasive ductal carcinoma (IDC) or invasive lobular carcinoma (ILC). The remaining 15% of cases include other types of BC with specific differentiation, including metaplastic carcinoma of the breast (MCB) [3].

The term MCB was introduced by Huvos et al. in 1973 [4]. MCB is characterized by a heterogeneous mixture of two or three histological lineages [5,6], including adenocarcinoma and other epithelial or mesenchymal components [7,8]. Among all of the BC types diagnosed annually, MCB represents [9] approximately 0.25-1% [8-19].

Generally, MCB exhibits no characteristic pattern in imaging studies, is characterized by rapid growth, and requires additional immunohistochemical (IHC) studies for its adequate characterization [5,8,11,12]. In most cases, MCB has a triple-negative (TN) IHC profile [20-25], resulting in a less-favorable prognosis [12,13,20,22,26]. Optimal management of a patient with a BC diagnosis depends on the patient’s clinical characteristics, histology, and the tumor’s IHC profile [22,27]. Management can include surgery and radiotherapy (RT) for local control [4,9,13]. The use of systemic chemotherapy (CT) has been reported with discouraging results [8,16,28]. The clinicopathologic characteristics and prognosis of MCB, compared with other, more common histologies, are poorly defined [7,8,24,29,30]. Some reports comparing the prognosis of MCB with those of other BC types suggest that MCB is more aggressive [7,12,22,24,29,31,32]. However, other reports have indicated that the prognosis of MCB is similar to that of adenocarcinoma [8,29].

This study aimed to determine whether an MCB diagnosis confers poor prognosis with respect to overall survival (OS) and disease-free survival (DFS) compared with other common histologies that are considered unfavorable (high-grade invasive ductal carcinoma [HG-IDC], triple-negative invasive ductal carcinoma (TN-IDC), and triple-negative invasive lobular carcinoma [TN-ILC]).

## Methods

Patients were selected from the database of the Mammary Tumors Department of the National Institute of Cancerology (INCan) in Mexico City between January 2005 and January 2013. We obtained the clinical file records of female patients diagnosed with MCB, IDC, or ILC using conventional histology by one of the authors. We excluded all patients with mixed histologies, those with a diagnosis of carcinoma *in situ*, those whose clinical file was incomplete, and those who had another histology or synchronous tumors in another organ.

From the IDC patient group, we created two subgroups, one defined as HG-IDC, to which patients with Scarff-Bloom-Richardson grading system 8 and 9 were assigned, and another group who had an IHC report of TN (estrogen receptors [ER] and progesterone receptors [PR] with nuclear staining of <1%, human epidermal receptor [HER2/neu] with IHC of ≤1, or fluorescence *in situ* hybridization at a ratio of HER2:centromere enumerator probe [CEP] 17 of <1.8). We paired subjects by age and clinical stage (CS), selecting two subjects for each type of MCB case. From the ILC patient group, we included classic and pleomorphic variants and selected patients with an IHC report of TN as previously described.

We obtained and analyzed the following variables: age; tumor size; lymph node status; distant metastasis; CS; the presence of hormonal receptors (HRs) and HER2/neu; use of induction therapy; pathological response; surgery performed; adjuvant therapy; and recurrence, progression and survival of the patients through the last follow-up appointment or date of death. For this final variable, we located patients or spoke with a family member by telephone to determine the patient’s status.

We conducted descriptive statistics for the demographic variables and report central tendency measures. We conducted univariate analysis of means using Student’s *t* test for continuous variables and the chi-square or Fisher exact test for ordinal variables.

We calculated the follow-up of each patient. DFS was defined as the period between the date at which the patient’s initial multimodal treatment (surgical, radiotherapeutic, and/or systemic) terminated and the date of recurrence or the patient’s last appointment. Recurrence was defined as the presence of disease >6 months after final treatment. Progression was defined as the presence of disease 6 months before treatment was finalized. OS was defined as the period between the date treatment ended and the date of the patient’s last appointment or death.

DFS and OS were analyzed by the Kaplan-Meier survival curve method, and the curves were compared using the log-rank test. We performed multivariate analysis to ascertain which variables exerted an influence on OS and DFS using the Cox proportional hazards model. Statistical significance was defined as a *p* value of <0.05. We used the statistical software program SPSS^®^ 2012 for Windows (SPSS Inc., Austin, TX, USA) for data analysis. The Director of Research of the INCan gave ethical approval for the collection and use of the data for this study.

This study was reviewed and approved by the Institutional Review Board of the National Cancer Institute of Mexico (approval number REV/20/14), and has been performed in compliance with the Helsinki Declaration. Since this study collected only archived data and materials, without using personal data from patients, we were not required by our federal legislation to obtain an informed consent from patients, as well as for phone calls, when preservation of confidentiality is assured. During the telephone call, the patient or the family member that answered was informed that a retrospective analysis was being performed and that the actual status was necessary for completing the information, also was established that all information will be kept confidential in accordance to the legislation and will be used only for this evaluation.

## Results

We identified 32 (0.6%) patients with a diagnosis of MCB among 5,440 patients diagnosed with BC. Of these 32 patients, we excluded 8 due to incomplete clinical records or having mixed histologies, resulting in a total of 24 patients for the final analysis. Of a total of 487 patients with an ILC diagnosis, we obtained 37 (7.6%) patients with TN-ILC. The patients with MCB were paired by age and CS at a ratio of 1:2 with patients with HG-IDC and TN-IDC, resulting in 48 patients for each group. All of the patients’ general clinicopathological characteristics are described in Table 1. We analyzed 157 patients in total, with an average follow-up of 40 months (range, 2.3-97.5 months).

**Table 1:** **Clinical and pathological characteristics of patients**

		MCB*n*(%)	TN-ILC*n*(%)	HG-IDC*n*(%)	TN-IDC*n*(%)	*p*value
	Number of cases	24	37	48	48	
Age	Mean	49.58	53.49	49.25	49.52	0.478
CS	I	0	0	0	0	0.364
	IIA	3 (12.5)	4 (10.8)	6 (12.5)	6 (12.5)	
	IIB	6 (25.0)	1 (2.7)	12 (25.0)	12 (25.0)	
	IIIA	6 (25.0)	13 (35.1)	12 (25.0)	12 (25.0)	
	IIIB	6 (25.0)	7 (18.9)	12 (25.0)	12 (25.0)	
	IIIC	1 (4.2)	6 (16.2)	2 (4.2)	2 (4.2)	
	IV	2 (8.3)	6 (16.2)	4 (8.3	4 (8.3)	
T	T1	0	0	0	0	0.114
	T2	0	3 (8.1)	1 (2.1)	1 (2.1)	
	T3	9 (37.5)	11 (29.7)	17 (35.4)	19 (39.6)	
	T4	15 (62.5)	23 (62.2)	30 (62.5)	28 (58.3)	
N	N0	7 (29.2)	5 (13.5)	9 (18.8)	8 (16.7)	0.748
	N1	8 (33.3)	10 (27.0)	19 (39.6)	18 (37.5)	
	N2	7 (29.2)	16 (43.2)	17 (35.4)	19 (39.6)	
	N3	2 (8.3)	6 (16.2)	3 (6.3)	3 (6.3)	
M	M0	22 (91.7)	28 (75.7)	44 (91.7)	44 (91.7)	0.323
	M1	2 (8.3)	9 (24.3)	4 (8.3)	4 (8.3)	
HR	Estrogen	2 (8.3)	---	29 (60.4)	---	<0.001
	Progesterone	3 (12.5)	---	26 (54.2)	---	<0.001
HER2/neu	Positive	1 (4.2)	---	14 (29.2)	---	<0.001

MCB: metaplastic carcinoma of the breast; TN-ILC: triple-negative invasive lobular carcinoma; HG-IDC: high-grade invasive ductal carcinoma; TN-IDC: triple-negative invasive ductal carcinoma; CS: clinical stage; T: tumor size; N: lymph nodes; M: distant metastasis; HR: hormone receptors; HER2/neu: human epidermal growth factor receptor 2; BC: breast cancer.

The 24 patients with a diagnosis of MCB had an average follow-up of 32.8 months (range, 4.1-73.4 months). The mean patient age was 49.6 years (range, 33-86 years). Three patients (12.5%) were grade IIA, 6 (25%) each were grade IIB, IIIA, and IIIB, 1 patient (4.2%) was grade IIIC, and 2 patients (8.3%) were grade IV. All patients had tumors >2 cm in size. Seven patients (29%) had no axillary lymph node metastases, and 2 patients (8.3%) had distant metastasis. The 48 HG-IDC patients had a mean follow-up of 48.3 months (range, 10.5-93.8 months), and the 48 TN-IDC patients had an average follow-up of 45.2 months (range, 5.0-97.5 months). The 37 patients diagnosed with TN-ILC had a mean follow-up of 34 months (range, 2.3-82.2 months) and a mean age of 53.49 years (range, 29-86 years). Table 2 presents the general characteristics of the treatment and clinical response.

**Table 2:** **Characteristics of treatment and response by histology**

		MCB*n*(%)	TN-ILC*n*(%)	HG-IDC*n*(%)	TN-IDC*n*(%)	*p*value
	Amount received	24	37	48	48	
Ind CT		12 (50)	20 (54.1)	38 (79.2)	39 (81.3)	<0.003
Response	pPR	2 (16.7)	15 (75)	26 (65)	24 (61.5)	<0.001
	pCR	3 (25.0)	2 (10.0)	12 (30)	11 (28.2)	
	Stable	1 (8.3)	1 (5)	0	2 (5.1)	
	Progression	6 (50)	2 (10)	2 (5)	2 (5.1)	
Surgery	BCS	0	0	3 (6.3)	7 (14.6)	0.011
	MRM	22 (91.7)	24 (64.9)	38 (79.2)	37 (77.1)	
	No	2 (8.3)	13 (35.1)	7 (14.6)	4 (8.3)	
Adjuvancy	Received	14 (58.3)	26 (70.3)	37 (77.1)	29 (60.4)	0.245
	CT	11 (45.8)	22 (59.5)	19 (39.6)	36 (75.0)	0.009
	RT	14 (58.3)	22 (59.5)	23 (47.9)	27 (56.3)	0.705
Recurrence	No	15 (62.5)	13 (35.1)	28 (58.3)	29 (60.4)	0.094
	Recurrence	3 (12.5)	12 (32.4)	18 (37.5)	16 (33.3)	
	Progression	6 (25)	12 (32.4)	2 (4.2)	3 (6.3)	

MCB: metaplastic carcinoma of the breast; TN-ILC: triple-negative invasive lobular carcinoma; HG-IDC: high-grade invasive ductal carcinoma; TN-IDC: triple-negative invasive ductal carcinoma; Ind CT: induction of chemotherapy; pPR: pathologic partial response; pCR: pathologic complete response; BCS: breast-conserving surgery; MRM: modified radical mastectomy; CT: adjuvant chemotherapy; RT: radiotherapy.

Tables 1 and 2 show the comparative analysis of the four histologies. The presence of HR and HER2/neu was more common in HG-IDC compared with MCB (ER: 60.4 vs. 8.3%; *p* <0.001; PR: 54.2 vs. 12.5%; *p* <0.001; HER2/neu: 29.2 vs. 4.2%; *p* <0.001). TN-IDC and HG-IDC patients were more likely to receive induction CT compared with TN-ILC and MCB patients (81.3, 79.2, 54.1, and 50%, respectively; *p* = 0.003). Regarding the type of pathological response, pathologic partial responses (pPR) were achieved by 75%, 65%, 61.5%, and 16.7% of TN-ILC, HG-IDC, TN-IDC, and MCB patients, respectively (*p* <0.001); similarly, pathologic complete responses (pCR) were less common in TN-ILC patients (10%) compared with HG-IDC (30%), TN-IDC (28.2%), and MCB patients (25%). Patients with MCB were more likely to progress during treatment compared with TN-ILC, TN-IDC, and HG-IDC patients (50%, 10%, 5.1%, and 5%, respectively*; p* <0.001). Among the MCB patients who experienced progression during CT induction, 4 received 5-fluorouracil-adriamycin-cyclophosphamide (FAC) plus paclitaxel and cisplatin as a radiosensitizer plus radiotherapy; 2 received only FAC and paclitaxel. In comparison, the patients who experienced partial and complete pathological responses were treated with FAC and paclitaxel. MRM was the most frequently performed surgical procedure and was performed in 91.7% of MCB patients, 79.2% of HG-IDC patients, 77.1% of TN-IDC patients, and 64.9% of TN-ILC patients (*p* = 0.011). Patients with TN-IDC received more adjuvant CT compared with TN-ILC, MCB, and HG-IDC patients (75%, 59.5%, 45.8%, and 39.6%, respectively; *p* = 0.009). With respect to RT, no differences were observed between MCB (32.4%), HG-IDC (47.9%), TN-IDC (56.3%) and TN-ILC patients (59.5%) (*p* = 0.705). In the MCB group, 3 patients (12.5%) experienced recurrence, 2 visceral and 1 to bone, whereas 6 patients (25%) experienced progression, 5 visceral and 1 loco-regional.

**Figure 1:**
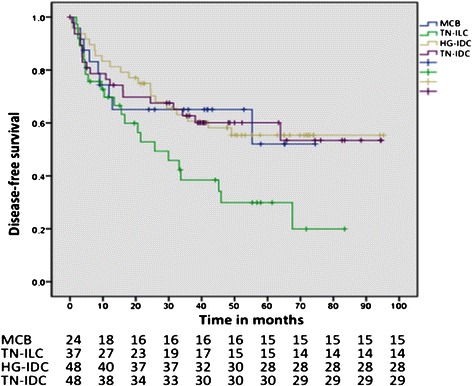
**Disease-free survival (DFS) by individual histology.** Metaplastic carcinoma of the breast (MCB) vs. high-grade invasive ductal carcinoma (HG-IDC), *p* = 0.865. MCB vs. triple-negative invasive ductal carcinoma (TN-IDC), *p* = 0.966. MCB vs. triple-negative invasive lobular carcinoma (TN-ILC)*, p* = 0.132.

**Figure 2:**
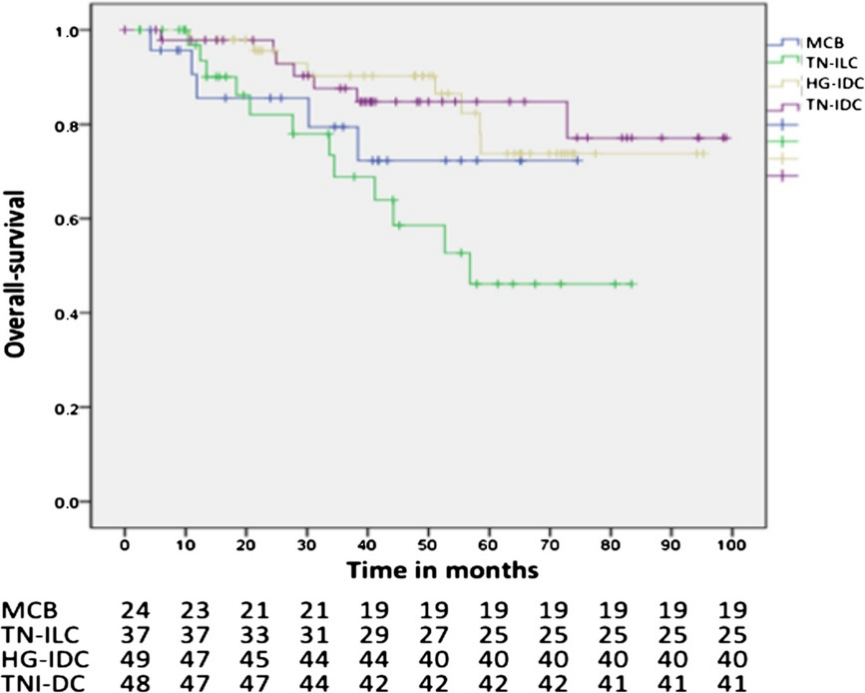
**Overall survival (OS) by individual histology.** Metaplastic carcinoma of the breast (MCB) vs. high-grade invasive ductal carcinoma (HG-IDC), *p* = 0.246. MCB vs. triple-negative invasive ductal carcinoma (TN-IDC), *p* = 0.255. MCB vs. triple-negative invasive lobular carcinoma (TN-ILC)*, p* = 0.387.

In patients with MCB, the 5-year DFS was 52.1% (mean, 48.52 months; 95% CI: 35.32-61.72 months), whereas the 5-year DFS was 55.4% for HG-IDC patients (mean, 61.52 months; 95% CI: 50.2-72.85 months), 60.1% for TN-IDC patients (mean, 59.81 months; 95% CI: 47.78-72.85 months), and 29.9% for TN-ILC patients (mean, 36.13 months; 95% CI: 25.33-46.93 months) (Table 3). When DFS was compared among all of the histologies, no significant differences were observed (*p* = 0.071) (Figure 1). The five-year OS was 72.2% for MCB patients (mean, 59.77 months; 95% CI: 48.55-71.00 months), 73.7% for HG-IDC patients (mean, 81.68 months; 95% CI: 73.38-89.98 months), 84.8% for TN-IDC patients (mean, 85.87 months; 95% CI: 77.10-94.64 months), and 44.3% for TN-ILC patients (mean, 56.68 months; 95% CI: 45.63-67.74 months) (Table 3). When the OS of all of the histologies was compared, significant differences were observed (*p* = 0.027). However, when the OS of MCB was compared with each of the histologies, no significant differences for any of the comparisons were detected (MCB vs. HG-IDC*, p* = 0.246; MCB vs. TN-IDC, *p* = 0.255, and MCB vs. TN-ILC, *p* = 0.387) (Figure 2).

**Table 3:** **Univariate analysis of means of overall survival (OS) and disease-free survival (DFS) by histology**

	MCB	TN-ILC	HG-IDC	TN-IDC	General	*p value*
OS in months (95% CI)	59.77 (48.55-71.00)	56.68 (45.63-67.74)	81.68 (73.38-89.98)	85.87 (77.10-94.64)	78.66 (72.64-84.68)	0.027
DFS in months (95% CI)	48.52 (35.32-61.72)	36.13 (25.33-46.93)	61.52 (50.20-72.85)	59.81 (47.78-71.84)	55.56 (48.86-62.26)	0.071

95% CI: 95% confidence interval; MCB: metaplastic carcinoma of the breast; TN-ILC: triple-negative invasive lobular carcinoma; HG-IDC: triple-negative invasive ductal carcinoma; TN-IDC: triple-negative invasive ductal carcinoma, OS: overall survival; DFS: disease-free survival.

We conducted a multivariate analysis with using a Cox proportional hazards model. In the comparative analysis, a significant difference (*p* <0.05) was observed, and none of the variables independently influenced OS or DFS.

## Discussion

Presently, BC is the most common neoplasm diagnosed in Mexico [2]; thus, infrequent histologies are observed more frequently than in other countries. One such histological type is MCB, and little is known about the clinicopathological characteristics and prognosis of MCB. Table 4 depicts the most representative series comparing MCB with other histologies.

**Table 4:** **Comparison with related studies**

Author	Histology	Age (years)	CS %	TN %	QX %	CT %	RT %	5-year OS	5-year DFS
Jung et al. (2010)	MCB *n* = 35	47.4	I 17.1 II 60.0 III 8.6 IV 8.6 X 1.0	80.0	MRM 48.6 BCS 51.4 No 0.0	88.6	71.4	62.8	41.8
	IDC *n* = 2,839	48.3	I 41.7 II 42.6 III 13.7 IV 2.0 X 0.0	16.7	MRM 24.3 BCS 74.8 No 1.0	80.6	82.1	92	87.3
	TN-IDC *n* = 473	48.1	I 40.4 II 46.5 III 11.2 IV 2.1 X 0.0	NA	MRM 31.7 BCS 67.7 No 0.6	90.5	84.1	83.6	81.8
Bae et al. (2011) [33]	MCB *n* = 47	47.0	I 23.4 II 70.2 III 6.4	93.6	MRM 21.3 BCS 78.7	89.4	80.9	NA	3 years 78.1
	IDC *n* = 1,346	48.6	I 37.8 II 47.2 III 15.0	16.2	MRM 41.0 BCS 59.0	81	67.4	NA	3 years 91.9
	TN-IDC *n* = 218	47.9	I 32.6 II 54.6 III 12.8	NA	MRM 33.9 BCS 66.1	90.9	73.9	NA	3 years 84.9
Song et al. (2013) [32]	MCB *n* = 55	≤50 = 49.1% >50 = 50.1%	I 7.3 II 54.6 III 29.1 X 9.1	67.3	MRM 92.7 BCS 7.7	48	49.1	54.5	45.5
	IDC *n* = 767	≤50 = 52.8% >50 = 47.2%	I 14.0 II 54.6 III 29.1 X 6.7	18.4	MRM 82.5 BCS 17.5	81.7	23.1	85.1	71.2
	TN-IDC *n* = 131	≤50 = 58.0% >50 = 42.0%	I 16.0 II 70.2 III 6.11 X 7.6	NA	MRM 80.9 BCS 19.1	80.9	27.1	73.3	60.3
Nelson et al. (2014) [38]	MCB *n* = 1,011	61	I 26.4 II 64.2 III 9.4	ER/PR (-) 77.8%	MRM 53.0 BCS 47.0	NA	48.3	71 *71	NA
	IDC *n* = 253,818	59	I 56.1 II 35.7 III 8.2	ER/PR (-) 21.3%	MRM 38.3 BCS 61.7	NA	54.3	88 *81	NA
Wright et al. (2014) [39]	MCB *n* = 2,338	NA	I 25.6 II 55.7 III 12.6 IV 6.0	ER/PR (-) 79.0%	NA	NA	NA	62.2 *63.5	NA
	IDC *n* = 382,667	NA	I 52.2 II 30.7 III 12.7 IV 4.3	ER/PR (-) 22.2%	NA	NA	NA	81.2 *72.2	NA
	ILC *n* = 44,813	NA	I 41.9 II 33.1 III 18.8 IV 6.3	ER/PR (-) 3.8%	NA	NA	NA	80.2 *59.5	NA
Barquet et al. (Current study)	MCB *n* = 24	49.6	II 37.5 III 54.2 IV 8.3	83.6	MRM 91.7 BCS 0.0 No 8.3	ind CT 50.0 adj CT 45.8	58.3	72.2	52.1
	HG-IDC *n* = 37	49.3	II 37.5 III 54.2 IV 8.3	40.0	MRM 79.2 BCS 6.3 No 14.6	ind CT 79.2 adj CT 39.6	47.9	73.7	55.4
	TN-IDC *n* = 48	49.5	II 37.5 III 54.2 IV 8.3	NA	MRM 77.1 BCS 14.6 No 8.3	ind CT 81.3 adj CT 75	56.3	84.8	60.1
	TN-ILC *n* = 48	53.5	II 13.5 III 70.2 IV 16.2	NA	MRM 64.9 BCS 0.0 No 35.1	ind CT 54.1 adj CT 59.5	59.5	44.3	29.9

CS: clinical stage; TN: triple negative; QX: type of surgery; CT: chemotherapy; RT: radiotherapy; OS: overall survival; DFS: disease-free survival; MCB: metaplastic carcinoma of the breast; IDC: invasive ductal carcinoma; TN-IDC: triple-negative invasive ductal carcinoma; ILC: invasive lobular carcinoma; ER/PR(-): negative estrogen and progesterone receptors; HG-IDC: high-grade invasive ductal carcinoma; TN-IDC: triple-negative invasive ductal carcinoma; TN-ILC: triple-negative invasive lobular carcinoma; X: not known; NA: not applicable; MRM: modified radical mastectomy; BCS: breast conservative surgery; ind CT: induction chemotherapy: adj CT: adjuvant chemotherapy; *OS in ER(-)/PR(-).

In this study, MCB represented 0.6% of all BC diagnosed, a percentage similar to those reported in other studies [5,7,10,11,22]. In our series, the mean age at diagnosis of MCB was >40 years, similarly to other studies [5,7,8,10-12,22,24,30]. All of the MCB and other BC patients exhibited tumors >5 cm. Furthermore, lymph node metastasis was observed in the majority of cases, and no difference in the rate of lymph node metastasis was observed with respect to other histologies. This finding can be explained by the nature of the study and the fact that Mexican female patients tend to present at an advanced stage. Previous studies have established that MCB tends to be diagnosed with larger tumors [7,22]. However, Bae et al. found no difference in tumor size between MCB and TN-IDC (*p* = 0.144) [33]. Lymph node metastasis has been shown to be less frequent in MCB [31,32], but not all studies have confirmed this observation [22,33]. In our study, 8.3% of the patient with MCB exhibited distant metastasis. However, our results with respect to MCB are consistent with the literature [22,31,34].

MCB was less likely to exhibit HR expression, with expression detected in 0-17% of cases [15,16,22]. Mourad et al. suggested that the absence of HR in MCB is due to the absence of an extensive glandular component [35]. Additionally, HER2/neu overexpression has been described within a range of 4-16% [31,33]. In our study, most patients with MBC were HR-negative or HER2/neu-negative, unlike patients with HG-IDC. This description is consistent with that of other studies [12]. Park et al. described that 84% of MCB cases were TN, as were 20.1% of IDC cases (*p* <0.001) [34].

Due to the rarity of MCB, few studies have evaluated the response to induction therapy [12,28]. In a series of 39 patients in which 9 patients were given induction CT, 8 exhibited progression, and all died due to the disease [28]. In our study, only half of the MCB patients received CT induction. Of patients who had a response, 25% had a pCR; however, 50% experienced progression during induction. In our institution, most patients who experience progression during induction CT have received radiotherapy with cisplatin as a radiosensitizer. Importantly, most MCB patients who exhibited progression had been treated with FAC-paclitaxel and concomitant cisplatin and radiotherapy, indicating that this histology responds poorly to this systemic treatment. This response may be associated with the intrinsic characteristics of MCB. According to Weigelt, MCB can be molecularly classified as claudin-low and basal-like [36], implying a behavior and systemic therapy response similar to those of MBC and mesenchymal neoplasias [37].

The group in which more BCSs were conducted was the TN-IDC group, followed by the HG-IDC group; notably, neither MCB nor TN-ILC patients were submitted to BCS, likely because these patients were diagnosed with larger tumors and exhibited poorer responses to induction CT. Song et al. also reported more mastectomies in MCB patients relative to TN-IDC patients (92.7 vs. 80.92%; *p* = 0.054) [32]; however, Bae et al. did not observe this difference (78.7 vs. 66.1%; *p* = 0.09) [33]. Of the patients who received adjuvant chemotherapy, nearly 50% received CT. This percentage can be explained by the fact that the patients who received induction CT were not included (Table 2). The percentage of patients who received RT was similar, with a slightly higher trend in MCB and TN-ILC compared with HG-IDC and TN-IDC (Table 2), possibly because BCS was less frequent among patients with MCB and TN-ILC. There is controversy in the literature concerning whether patients with MCB tend to receive more RT than other histologies [31-33].

The mean DFS of MCB was 48.52 months, with a 5-year DFS of 52.1%. No significant differences were observed in DFS between MCB and each of the other histologies analyzed (Figure 2). The mean OS of patients with MCB was 59.77 months, with a 5-year OS of 72.2%. When comparing the OS of the groups studied, a difference was observed between the four groups (*p* = 0.027). However, when comparing the OS of MCB with each group, this difference was not detected (Figure 1). In patients with MCB, mean OS decreased to 40 months (72.2 and 73%, respectively); thus, patients with MCB stop dying, whereas patients with TN-ILC continue to do so (80-month OS of 72.2% vs. 44.3%, respectively) (Figure 1). Notably, in our study, both MCB and TN-ILC patients exhibited worse OS compared with TN-IDC patients, making both histological types a worse prognostic factor for the OS of patients with breast cancer. Whether histology modifies OS and DFS remains controversial. In the Bae et al. study, the 3-year DFS did not differ between patients with MCB and TN-IDC (78.1% vs. 84.9%; *p* <0.001) [3]. Lai et al. found that the 5-year DFS did not differ between MCB and ILC as much as the 5-year OS (*p* = 0.289 and 0.132, respectively) [31]. Song et al. reported that the prognosis of MCB was worse than that of TN-IDC, with a 5-year OS of 54.5% for MCB vs. 73.3% for TN-IDC (*p* <0.001) and a 5-year DFS of 45.5% in MCB vs. 60.3% in TN-IDC (*p* <0.001) [32]. Larger studies have been conducted to compare MCB and other histologies, including HR-negative patients with negative hormonal receptors, using patient information from the Surveillance, Epidemiology, and End Results from United States [38,39]. Nelson et al. compared 1,011 patients with MBC with 253,818 patients with IDC between 2001 and 2010; the authors also compared patients with respect to hormone receptors status and a matching analysis. The paper concludes that MBC conferred a worse OS and disease-specific survival (DSS) at 5 years compared with IDC (71% vs. 88%, and 78% vs. 93%, respectively), HR-negative cancers (71% vs. 81% and 77% vs. 85%, respectively), and the matched group (72% vs. 79% and 79% vs. 85%, respectively) [38]. The second study was performed by Wright et al., who compared 2,338 patients with MCB with 382,667 patients with IDC; additionally, 44,813 patients with ILC were included. This study reported that patients with MCB exhibited a worse OS compared with IDC (62.1% vs. 82.1%*, p* <0.001), including those who were HR-negative (63.5% vs. 72.2%, *p* <0001); however, when the authors compared MCB and ILC with negative receptors, no difference was detected with respect to OS (63.5% vs. 59.5%, *p* >0.15) and DSS (71.3% vs. 72.2%, *p* >0.65), similar to the TN-ILC and MCB findings in our study [39]. However, both studies compared cancers based only on the hormone receptors status, without knowing the HER2/neu status or type of CT applied.

To our knowledge, this is the first study on MCB reported in the literature in Latin America and particularly in a Mexican population; furthermore, this is the first study to compare MCB with TN-ILC. Our study has several limitations, such as its retrospective design, the number of MCB cases, the focus only on IHC characteristics, and the relatively short mean follow-up. Therefore, future studies should consider the molecular characteristics of each of the tumors.

## Conclusions

MCB is an infrequent entity and thus is rarely studied. MCB tends to exhibit less BCS, likely due to the CS at which it is diagnosed, its TN receptors and because it exhibits disease progression. However, our study demonstrated that the OS and DFS in patients with MCB do not differ from those of patients with the most common BC histologies with poor IHC profiles. Future studies should determine whether the molecular characteristics contribute to the prognosis of this type of BC.

## Abbreviations

MCB: Metaplastic carcinoma of the breast
OS: Overall survival
DFS: Disease-free survival
TN-ILC: triple-negative invasive lobular carcinoma
HG-IDC: High-grade invasive ductal carcinoma
TN-IDC: Triple-negative invasive ductal carcinoma
BC: Breast cancer
IDC: Invasive ductal carcinoma
ILC: Invasive lobular carcinoma
IHC: Immunohistochemical
TN: Triple negative
RT: radiotherapy
CT: Chemotherapy
INCan: National Institute of Cancerology
ER: Estrogen receptors
PR: Progesterone receptors
HER2/neu: Human epidermal receptor
CS: Clinical stage
HR: Hormonal receptors
pPR: Pathologic partial response
pCR: Pathologic complete response
